# Exploring the mediating role of promoting school physical activity on the relationship between low socioeconomic status and academic achievement and school climate: evidence from 4,990 Chilean schools

**DOI:** 10.3389/fpubh.2024.1426108

**Published:** 2024-06-05

**Authors:** Pedro Delgado-Floody, Carlos Cristi-Montero, Daniel Jerez-Mayorga, Alberto Ruiz-Ariza, Iris Paola Guzmán-Guzmán, Cristian Álvarez, Manuel Gómez-López, Bastian Carter-Thuillier, Felipe Caamaño-Navarrete

**Affiliations:** ^1^Department of Physical Education, Sports and Recreation, Universidad de La Frontera, Temuco, Chile; ^2^IRyS Group, Physical Education School, Pontificia Universidad Católica de Valparaíso, Valparaíso, Chile; ^3^Department of Physical Education and Sports, Faculty of Sport Sciences, University of Granada, Granada, Spain; ^4^Exercise and Rehabilitation Sciences Institute, School of Physical Therapy, Faculty of Rehabilitation Sciences, Universidad Andres Bello, Santiago de Chile, Chile; ^5^Faculty of Educational Sciences, University of Jaén, Jaén, Spain; ^6^Faculty of Chemical-Biological Sciences, Universidad Autónoma de Guerrero, Chilpancingo, Guerrero, Mexico; ^7^Department of Physical Activity and Sport, Faculty of Sports Sciences, University of Murcia, Santiago de la Ribera, Murcia, Spain; ^8^Department of Education, Universidad de Los Lagos, Osorno, Chile; ^9^Programa de Investigación en Deporte, Sociedad y Buen Vivir, Universidad de Los Lagos, Osorno, Chile; ^10^Departamento de Didáctica y Práctica, Facultad de Educación, Universidad Católica de Temuco, Temuco, Chile; ^11^Physical Education Career, Universidad Autónoma de Chile, Temuco, Chile

**Keywords:** physical activity, academic performance, socioeconomic status, school climate, children

## Abstract

There is evidence that promoting school physical activity (PSPA) benefits children and adolescents, but little is understood about how this promotion may relate to academic achievement and school climate across varying levels of socioeconomic status (SES). Hence, the study aimed to address this knowledge gap by examining two main objectives: (1) determining the association between PSPA and academic achievement and school climate according to schools’ SES and (2) exploring the potential mediating role of PSPA in the relationship between schools’ SES and academic achievement and school climate. This cross-sectional study at the school level focused on 4,990 schools (including public, subsidized, and private schools) that participated in the National Educational Study 2018 (Chile), which was applied to primary schoolchildren (4th grade, aged 8–10 years). Schools were divided into non-PSPA (*n* = 4,280) and PSPA (*n* = 710) during the year 2018. Changes in academic achievement from 2017 to 2018 and school climate were considered. PSPA was associated with improvements in maths (low-SES OR: 1.80, *p* < 0.001) and reading (middle-SES OR: 1.45, *p* = 0.029; low-SES OR: 1.47, *p* < 0.001). The indirect effect (IE) showed that PSPA partially mediated the relationship between SES and academic achievement in reading (IE = 1.017; SE = 0.12; 95%CI, −1.27, −0.77), maths (IE = –1.019; SE = 0.12; 95%CI, −1.25, −0.78), and school climate (IE = –0.46; SE = 0.52; 95%CI, −0.56, −0.35). In conclusion, PSPA was linked to positive changes in academic achievement, especially among low SES, and PSPA presented a potential mediating role in the relationship between SES of schools and academic achievement and school climate.

## 1 Introduction

### 1.1 Socioeconomic status

Low socioeconomic status (SES) has been associated with poor academic achievement in different countries around the world, particularly in developing countries (1, 2). In schools where high SES predominates among the student cohort, children typically report superior levels of academic achievement compared to children attending schools where low SES predominates (2, 3). Such findings are concerning because they reveal significant structural social differences and segregation and also highlight the severe challenges that educational institutions face in overcoming patterns of reproduction and providing increased opportunities for citizens to become upwardly mobile.

Chile is relevant in this sense because it has among the highest levels of economic and social inequalities in the world (4). Although it has experienced significant economic growth in the past decades, inequalities in accessing a quality education among all sectors of the population have not been overcome (5). Additionally, Chile has one of the highest Gini coefficients among Organization for Economic Co-operation and Development (OECD) countries (6) and it is well established that the Chilean national educational system is highly segregated (7). In this respect, SES segregation is a central problem for the country’s educational system. Specifically, there is a significant contrast in the results attained by students at private and public schools, a situation that has been strongly criticized, as it can perpetuate the existing educational, economic, and social gap (8). In fact, previous data regarding Chilean students indicate that family income is a strong predictor of academic achievement (9). Furthermore, students who come from families with higher poverty rates mostly attend public schools, whose students generally perform more poorly in the main national tests than their counterparts at private schools with superior SES (10). Therefore, in order to achieve social progress, it is necessary to find strategies aimed at closing this gap between private and public schools.

### 1.2 School climate

Similarly, in the Chilean context, it has been shown that school climate has a direct relationship with the prevailing SES: schools whose students are primarily of low SES tend to have an unhealthy school climate (3), which affects social and emotional interactions and social relationships of school children with their teachers (11), thereby exerting a direct impact on their academic achievement (12). In other words, a healthy school climate can promote and facilitate learning among schoolchildren.

Accordingly, a school climate built on reciprocity—including the existence of a respectful atmosphere and supportive relationships among all stakeholders—can enable students to develop socially, emotionally, and academically as well as improve their predisposition to learn (12). Therefore, educational policies must approach the relationship between poverty, school climate, and academic achievement.

Existing evidence (13–16) suggests that improvements in the climate of a school can disrupt the negative link between low SES and poor academic achievement in various contexts, rendering it a key factor in promoting equal educational opportunities, which should be understood as a duty of all school community members, not only students, in order to promote high socio-normative expectations in schools. A recent study conducted with Chilean students indicated that a positive classroom climate compensates for the negative effects of low SES on SIMCE scores, i.e., maths tests (17). Moreover, a systematic review (18) focusing on school climate and children’s academic and psychological wellbeing highlighted the importance of a positive relationship between these variables in order to ensure effective learning, especially in impoverished contexts, where improving relationships between students and other school members can have a significant impact on academic achievement. In addition, school climate can not only benefit academic performance but has also been linked with socio-emotional health among schoolchildren (19). Another study reported that improvements in school climate can increase students’ academic opportunities, especially in schools with large proportions of low-SES families (13).

### 1.3 Promoting school physical activity

Physical activity (PA) may have a beneficial impact on academic achievement as well as on school climate; in concrete terms, different studies have reported that PA patterns have a positive effect on academic achievement, cognitive development, and school climate (20, 21). Moreover, existing evidence suggests that higher levels of PA, especially in physical education (PE) classes, produce a better school climate in addition to enabling students to perform better academically, particularly in skills related to maths and reading (22). In addition, there is strong evidence of a positive link between PA and academic achievement (23). In this context, another study focused on the historical perspective of 125 published articles, indicating that PA had a positive effect on the academic achievement of schoolchildren (24). Furthermore, an umbrella review and meta-analysis study showed that regular PA had a medium positive effect on academic achievement, suggesting that PA can boost the academic achievement of children and adolescents (25). Accordingly, another study found that meeting PA recommendations was positively linked with better academic achievement (26). Other evidence (27–29) suggests that engaging in physical activity during school age has a positive impact on children’s physical, cognitive, academic, social, emotional, and motor development. Indeed, PA has proved to be a positive instrument for developing interpersonal relationships, fostering social inclusion processes, improving self-esteem and self-confidence, promoting social and emotional skills, and encouraging healthy lifestyles; hence, physical activity can help build a better school climate and improve learning processes. Similarly, a meta-analysis reported that PA interventions could constitute a promising strategy for improving cognitive outcomes and language skills (30).

In Chile, the results from Chile’s 2018 Report Card on Physical Activity for Children and Youth indicated that 20.2% of children and adolescents meet the PA guidelines. Specifically, 27.4% of children aged 9–11 years old and 18.9% of adolescents meet the PA guidelines (31). In this sense, the World Health Organization has proposed promoting physical activity through schools (32). Based on previously reported findings, interventions that promote school physical activity (PSPA) have been applied in the classroom to understand the immediate effect of PA on academic performance, obtaining positive results (33). For instance, a systematic review reported that PA interventions can have positive effects on students’ performance in maths tests (34). Similarly, PSPA through the academic curriculum has been well received and successful in improving academic achievement (35). In the Chilean educational system, for example, PSPA through sports and recreational programs in order to stimulate an active and healthy lifestyle (i.e., schools take greater responsibility for PSPA) has been linked to better academic achievement (3). In fact, existing evidence suggests that increasing the amount of time dedicated to physical education classes may help boost academic achievement (36). It has also been shown that PSPA can be beneficial in promoting the functionary and cognitive process development of the brain among school-age children (37).

According to our knowledge, the evidence has reported different benefits of developing PA, but there is a scarcity of studies involving schools of different SES promoting PA and how it could play some role in the academic and non-academic achievement of schoolchildren. Due to the importance of learning and a good school climate, this study aimed to address this knowledge gap by examining two main objectives: (1) determining the association between PSPA and academic achievement and school climate according to schools’ SES and (2) exploring the potential mediating role of PSPA in the relationship between SES of schools and academic achievement and school climate. The present study hypothesizes that PSPA is positively associated with academic achievement and school climate and presents a mediating role in the established relationship between SES of schools and academic achievement and school climate.

## 2 Materials and methods

### 2.1 Participants

This cross-sectional study at the school level focused on 4,990 schools (including public, subsidized, and private schools) that participated in the National Educational Study 2018 (Chile), which was applied to primary schoolchildren (4th grade, aged 8–10 years). The data from the year 2017 only considered academic achievement to determine delta changes (2017 vs. 2018 scores in reading and maths) during the year 2018, whereas the data from 2018 additionally considered the percentage of students for each school according to the achievement of academic standards (% insufficient, elemental or adequate) and school climate. PSPA was obtained in 2018 to classify the schools as either PSPA or non-PSPA. A total of 215,210 schoolchildren took the test in reading and 215,999 took the test in maths (reference value year 2018 data). The research team agreed to the ethical commitments of the Chilean state to protect the identities of schools and students and undertook to use the data solely for scientific purposes.

### 2.2 Selection of educational establishments

In the first step, the databases of the Agency of Quality Education (2018) of the Chilean Ministry of Education were reviewed by the research team, where 7,414 schools were detected according to the national codes register (RBD code); each school in Chile has a national register code. Subsequently, it was checked that they had their academic indicators of reading and mathematics, based on which 653 schools were excluded (*n* = 6,761). In the next step, the non-academic indicators of PSPA of schools and school climate were incorporated. Finally, 1,771 schools that did not present the academic standards in reading and mathematics were excluded (*n* = 4,990 final sample).

### 2.3 Measures

All the data used in this study were officially requested and obtained from the Agency of Quality Education, Ministry of Education in Chile. This state department formally granted all the necessary permits for the research team to use the data for research purposes and subsequent publication. The Ministry of Education applies standardized tests in all the schools of the country with the objective of assessing academic (reading and maths) and non-academic (PSPA and school climate) achievement. The averages of each school in this regard are reported by the Agency of Quality Education in its database and were used for this research.

#### 2.3.1 Academic achievement

Academic achievement was measured using scores in reading and maths of each school (which constitute part of the National Educational Study). The content of these tests is designed by government specialists and their use is confidential; in addition, all tests are multiple choice. In this research, each child’s academic scores in 2018 were considered in order to obtain each school’s average; these averages were then compared with those of the preceding year (2017) to determine the delta changes (∆ 2017 vs. 2018).

Moreover, the same test (2018) reported the academic standards describing what a student should know and be able to know based on the national school curriculum at the time. In Chile, academic standards are developed based on the current national school curriculum and are associated with the instrument by which their compliance is evaluated. The standards are classified as follows:

- *Insufficient learning standard*: students who are classified at this level fail to demonstrate consistently that they have acquired the most elementary knowledge and skills stipulated in the curriculum for the period evaluated.
- *Elementary learning standard*: students who reach this level of learning have partially achieved what is required in the curriculum.
- *Adequate learning standard*: students who reach this level of learning have achieved what is required in the curriculum in a satisfactory manner. This implies demonstrating that they have acquired the basic knowledge and skills stipulated in the curriculum for the evaluated period.

#### 2.3.2 Non-academic evaluation

Non-academic achievements of schools were self-reported by students, parents, teachers, and head teachers during the year 2018 (38). The content of these tests and questionnaires is designed by government specialists, and their use is confidential. Different indicators, such as PSPA and school climate, use some reference tests and are adapted by the Agency of Education Quality in Chile. Indicator scores are expressed on a scale from 0 to 100; each student is assigned a score based on their responses to indicator-related questionnaire questions. The scores of all students in a given grade who answer the questionnaire are averaged, and a score is assigned to their school.

#### 2.3.3 Promoting school physical activity

This outcome was used to evaluate the perception of schoolchildren and the Agency of Education Quality information according to the degree to which the school PSPA, health activities, and an active lifestyle. Moreover, self-declared attitudes and behaviors related to an active lifestyle and their perceptions of the PSPA of school in the school space considering sports, recreation, PA (different types), dance, and all kinds of programs or activities that promote PA and a healthy lifestyle. A previous national investigation in Chile reported good indicators of PSPA, with values greater than 70 points (3). The PSPA index was calculated according to the aforementioned study, which involved diverse sources (3). The PSPA index contains information from the California Healthy Kids Survey, the results of the Physical Education National Study (Chile) (39), and the administrative records of the Agency of Education Quality (38). Its scores range between 0 and 100 points; a score > 70 points is classified as PSPA (i.e., schools present greater responsibility for PSPA), whereas a score ≤ 70 points is classified as non-PSPA (3) (i.e., schools no present responsibility for PSPA).

#### 2.3.4 School climate

School climate was evaluated using the perceptions of students, teachers, and parents through interviews, focus groups, and self-reported surveys. These were designed to explore stakeholders’ perceptions and attitudes in three sub-dimensions: (a) respectful school climate; (b) organized environment; and (c) safe school environment. The questionnaires mentioned by the Agency of Education Quality in Chile are the following. (i) The School Climate Survey (40) is used to evaluate school climate, with the objective (referring to school coexistence) of inquiring into interpersonal treatment and security in an establishment, with special emphasis placed on bullying. (ii) The Comprehensive School Climate Inventory is used to provide information on how students, parents, teachers, and other members of the educational community perceive the school climate of their establishment. (iii) The Survey on School Coexistence for Students is an instrument that is only used in Chile and was originally created by the Citizen Peace Foundation in 2005. It consists of a questionnaire that evaluates students’ perceptions of situations of violence within the school.

#### 2.3.5 Socioeconomic status (SES)

School SES was determined through an integrative score calculated by the Ministry of Education which includes the following: (i) the educational level of a student’s mother and father; (ii) the total monthly income of the household; and (iii) the school vulnerability index (SVI). The SVI is an indicator that measures students’ vulnerability considering the school dropout risk and a complex socioeconomic evaluation of students and families. Through its use, an average value per establishment is obtained, falling into one of five categories (low, middle–low, middle, middle–high, and high SES). For the present study, three SES groups were computed: (A) low SES (comprising low- and middle–low SES); (B) middle SES (including middle SES); and (C) high SES (including middle–high and high SES).

### 2.4 Statistical analysis

The data are presented as mean and the standard deviation (SD). Normality and homoscedasticity assumptions for all the data were analyzed using the Kolmogorov–Smirnov test and the Levene test, respectively. Analysis of covariance (ANCOVA) was used to identify differences between groups (PSPA vs. non-PSPA) in order to compare differences among schools according to SES (as references and informative values), while the Bonferroni *post-hoc* test was applied to test differences among groups, as co-variables were considered school dependence and SES. Delta (∆) changes were estimated to compare academic achievement (2017 vs. 2018) according to PSPA and non-PSPA in different SES, with multivariate analysis with Šidák’s *post-hoc* test used for multiple comparisons and eta partial squared for interaction (Time [Year] x Group [Non-PSPA/PSPA], by different SES was assessed by *η*^2^ obtained with small (*η*^2^ = 0.01), medium (*η*^2^ = 0.06), and large (*η*^2^ = 0.14). A logistic regression was used to determine the association between changes in academic achievement (2017 vs. 2018) and PSPA. Overall, the alpha level was set at *p* < 0.05 for statistical significance.

Regression analyses were performed to verify the effect of the mediating variables PSPA (M), considering SES as the independent variable (X) and academic achievement and school climate as the dependent variables (Y). Within the analysis, the total effect (c), direct effect (c`), and indirect effect (a*b, IE) were calculated for the samples as well as the 95% confidence interval (CI) using the macro/interface PROCESS v. 3.3 for SPSS v. 23 and the bootstrapping method with a resampling rate of 5,000 (41). The indirect effect was considered significant if 0 was outside the 95% confidence interval. The percentage of mediation was estimated as a proportion between the direct and the total effect 1- (c´/c). All the statistical analyses were performed with SPSS statistical software version 23.0 (SPSS™ Inc., Chicago, IL). The alpha level was set at *p* < 0.05 for statistical significance.

## 3 Results

Table 1 shows the comparison of academic achievement, standard, and school climate according to PSPA and non-PSPA (the comparison of SES is presented as reference values). According to the delta changes in reading (non-PSPA: 1.90 ± 19.020 vs. PSPA: 6.50 ± 21.21 score, *p* < 0.001) and maths (non-PSPA: −1.66 ± 17.64 vs. PSPA: 3.34 ± 20.05 score, *p* < 0.001), schools that PSPA reported better results than those that did not. Indeed, the former reported a lower percentage of students reaching an insufficient academic standard in reading (non-PSPA: 33.06 ± 17.50 vs. PSPA: 25.55 ± 15.76%, *p* < 0.001) and maths (non-PSPA: 42.06 ± 21.55 vs. PSPA: 33.83 ± 20.71%, *p* < 0.001). The schools PSPA also reported better school climate (non-PSPA: 74.30 ± 5.01 vs. PSPA: 80.33 ± 4.67, *p* < 0.001) than the latter.

**Table 1:** Characteristics of the chilean schools and comparison according to promoting physical activity and socioeconomic status of schools in the year 2018.

		Promoting physical activity			Socioeconomic status (additional reference data)			
	Total (*n* = 4,990)	Non-promoting PA (*n* = 4,280)	Promoting PA (*n* = 710)	*p*-value	Low SES (*n* = 2,605) A	Middle SES (*n* = 1,444) B	High SES (*n* = 941) C	*p*-value
Academic achievement in reading					Academic achievement in reading			
Reading 2017 (score)	264.34 ± 24.67	263.56 ± 24.45	269.07 ± 25.49	*p* < 0.001	253.87 ± 21.89^B, C^	266.35 ± 19.34	290.25 ± 18.59	*p* < 0.001
Reading 2018 (score)	266.89 ± 23.51	265.46 ± 23.36	275.57 ± 22.59	*p* < 0.001	256.14 ± 20.54^B, C^	269.95 ± 18.05^C^	291.96 ± 17.37	*p* < 0.001
∆	2.55 ± 19.41	1.90 ± 19.020	6.50 ± 21.21	*p* < 0.001	2.28 ± 21.82^B^	3.60 ± 17.34^C^	1.71 ± 14.71^a,b^	*p* = 0.029
% Schoolchildren in different academic standards 2018					% Schoolchildren in different academic standards 2018			
Insufficient (% mean)	31.99 ± 17.46	33.06 ± 17.50	25.55 ± 15.76	*p* < 0.001	39.48 ± 16.84^B, C^	29.09 ± 13.75^C^	15.72 ± 10.42	*p* < 0.001
Elemental (% mean)	26.60 ± 9.17	26.54 ± 9.04	26.98 ± 9.92	*p* = 0.405	27.50 ± 9.82^C^	27.41 ± 8.00^C^	22.86 ± 7.99	*p* < 0.001
Adequate (% mean)	41.41 ± 18.40	40.40 ± 18.11	47.48 ± 18.95	*p* < 0.001	33.02 ± 15.47^B, C^	43.50 ± 14.53^C^	61.41 ± 14.38	*p* < 0.001
Academic achievement in math					Academic achievement in math			
Math 2017 (score)	255.92 ± 25.56	255.22 ± 25.42	260.17 ± 25.99	*p* < 0.001	245.33 ± 2.02^B, C^	57.87 ± 20.68^C^	282.27 ± 21.32	*p* < 0.001
Math 2018 (score)	254.97 ± 25.66	253.56 ± 25.47	263.52 ± 25.10	*p* < 0.001	244.32 ± 22.25^B, C^	257.02 ± 20.34^C^	281.32 ± 21.70	*p* < 0.001
∆	−0.95 ± 18.08	−1.66 ± 17.64	3.34 ± 20.05	*p* < 0.001	−1.01 ± 20.54^B, C^	−0.86 ± 15.84	−0.95 ± 13.48	*p* = 0.185
% Schoolchildren in different academic standards 2018					% Schoolchildren in different academic standards 2018			
Insufficient (% mean)	40.89 ± 21.62	42.06 ± 21.55	33.83 ± 20.71	*p* < 0.001	49.74 ± 20.06^B, C^	38.55 ± 17.59^C^	20.07 ± 15.26	*p* < 0.001
Elemental (% mean)	37.87 ± 11.92	37.53 ± 11.81	39.92 ± 12.38	*p* = 0.343	35.63 ± 12.80^B, C^	40.50 ± 9.80	40.03 ± 11.13	*p* < 0.001
Adequate (% mean)	21.24 ± 17.75	20.41 ± 17.31	26.25 ± 19.50	*p* < 0.001	14.64 ± 13.07^B, C^	20.96 ± 14.37^C^	39.90 ± 20.29	*p* < 0.001
Non-academic achievement					Non-academic achievement			
School climate (score)	75.16 ± 5.39	74.30 ± 5.01	80.33 ± 4.67	*p* < 0.001	74.31 ± 5.79^B, C^	75.58 ± 5.05^C^	76.87 ± 4.11	*p* = 0.001

The values shown are presented as mean ± SD; *p*-value < 0.05 is considered statistically significant adjusted by school dependence and SES in PSPA and adjusted by school dependence in SES comparison. SES = socioeconomic status. A, B, or C in the super index reported differences with this group. A = Low-SES, B = middle-SES, C = high-SES.

**Figure 1:**
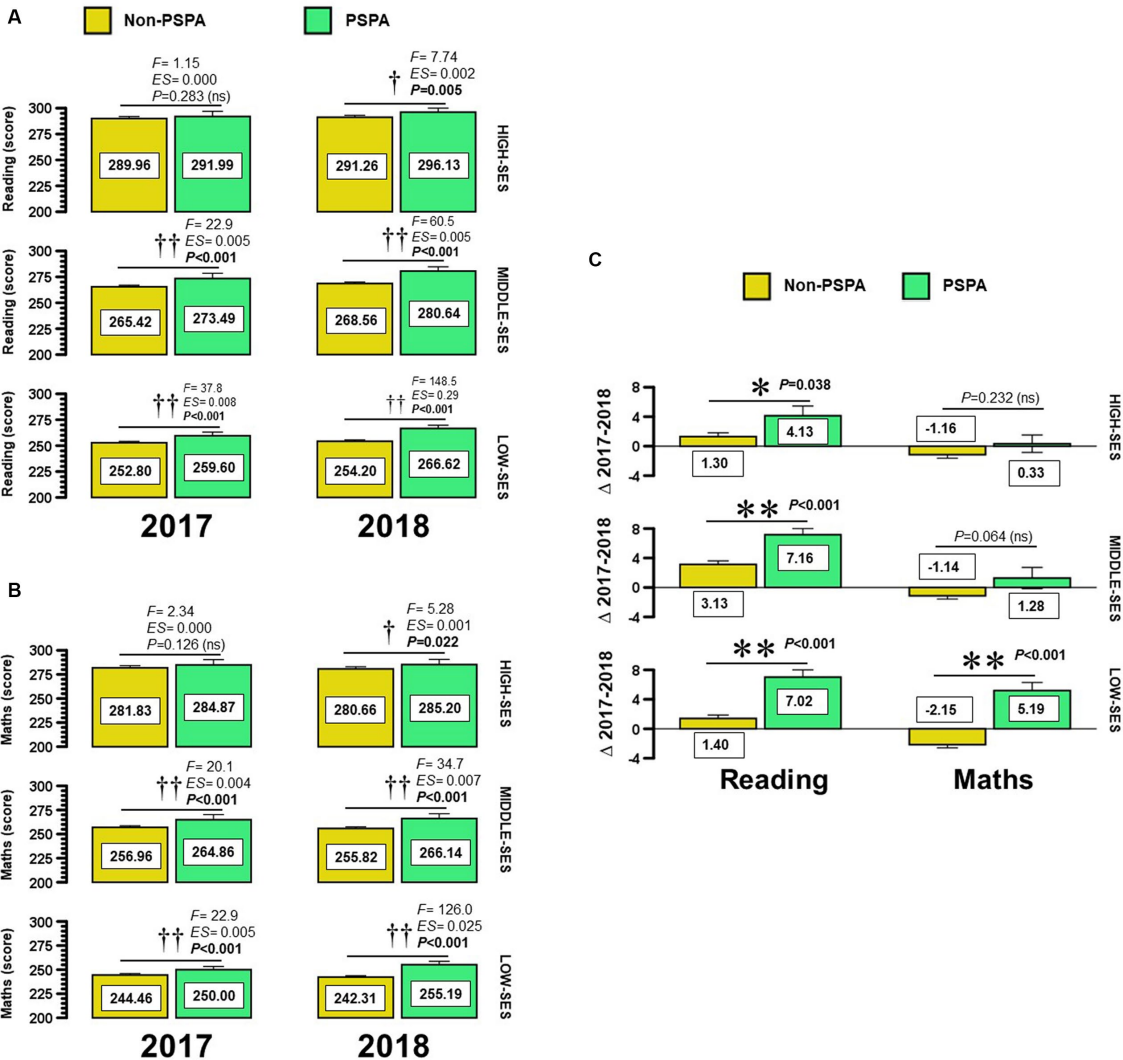
Changes from 2017 to 2018 year in the ‘Reading’ and ‘Maths’ score in Chilean students of primary school by schools of different socio-economic status. Panel **(A, B)** show absolute data from 2017 to 2018, and panel **(C)** the respective delta changes (∆) to both ‘Reading’ and ‘Math’ score. Categories are shown as Non-Promoting Physical Activity (Non-PSPA) and Schools that Promoting School Physical Activity (PSA). (High-SES) High socio-economic status, (Middle-SES) Middle socio-economic status, and ((Low-SES) Low socio-economic status. (F) Levene test, and (ES) Laken’s effect size. (†) Denotes significant differences between Non-promoting PA versus Promoting PA at *P*<0.05, and (††) at *P*<0.001. (*) Denotes significant differences between 2017 versus 2018 year at *P*<0.05, and (**) at *P*<0.001..

Figure 1 shows the comparison of changes in academic achievement during the year 2018 (in comparison with 2017) according to PSPA in each school by SES. Low-SES schools reported larger differences in reading (non-PSPA, ∆: 1.40 ± 21.45 vs. PSPA ∆: 7.02 ± 23.18, *p* = 0.001) (Figure 1C) and maths (non-PSPA ∆: −2.15 ± 20.04 vs. PSPA ∆: 5.19 ± 22.06, *p* = 0.001) (Figure 1C). Among middle-SES schools, PSPA schools reported better results than non-PSPA schools in reading (non-PSPA ∆: 3.13 ± 16.88 vs. PSPA ∆: 7.16 ± 20.24, *p* = 0.005). Among high-SES schools, PSPA schools reported larger changes in reading (non-PSPA ∆: 1.30 ± 14.57 vs. PSPA ∆: 4.13 ± 15.35, *p* = 0.038) (Figure 1C).

**Figure 2:**
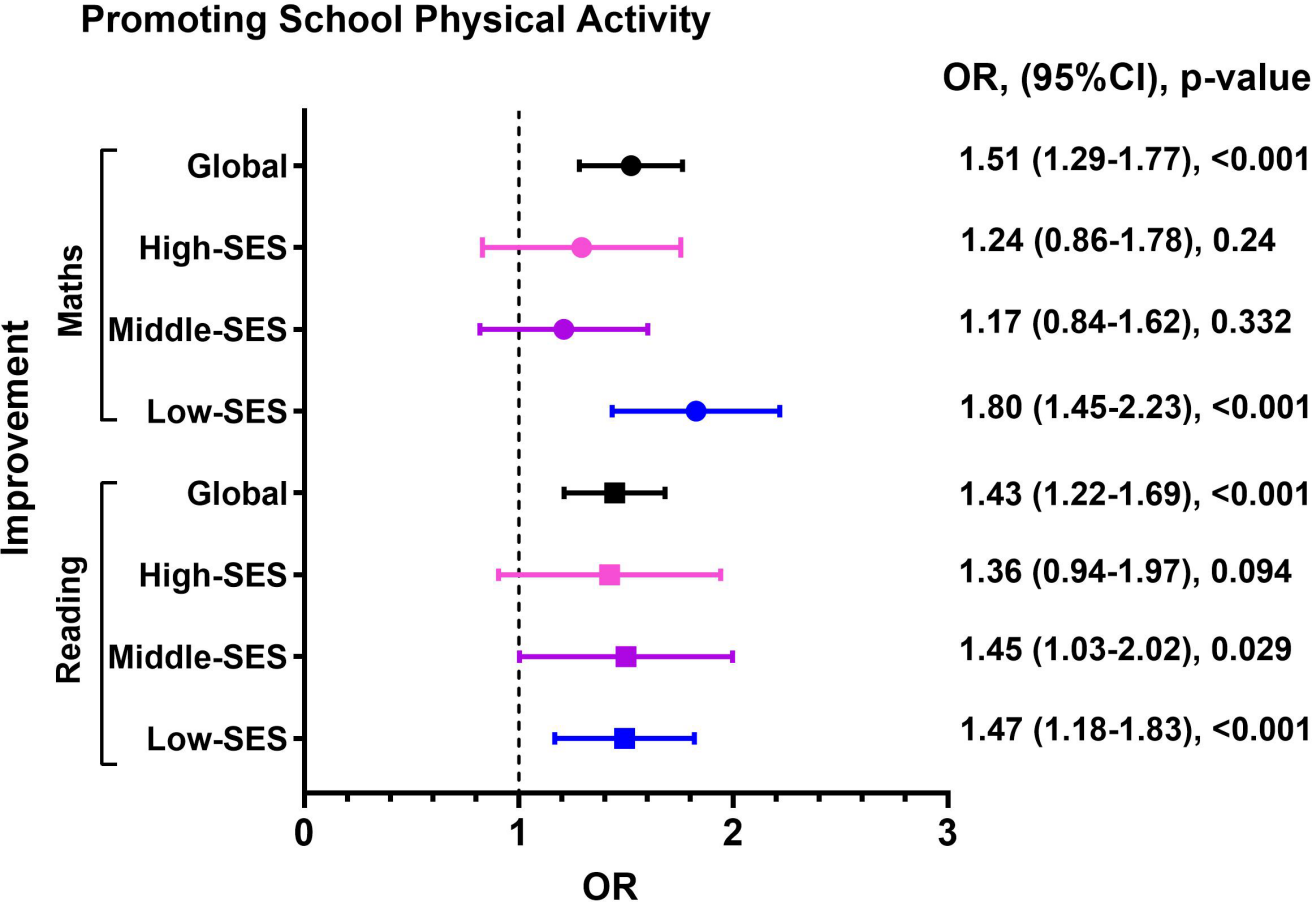
Association between improvement academic achievement with Promoting School Physical Activity according Schools-SES.

PSPA showed a positive association with improvements in maths in low-SES schools (OR; 1.80, 95%CI; 1.45–2.23, *p* < 0.001). It was also linked to improvements in reading in middle SES (OR; 1.45, 95%CI; 1.03–2.02, *p* = 0.029) and low-SES schools (OR; 1.47, 95%CI; 1.18–1.83, *p* < 0.001) (Figure 2).

**Figure 3:**
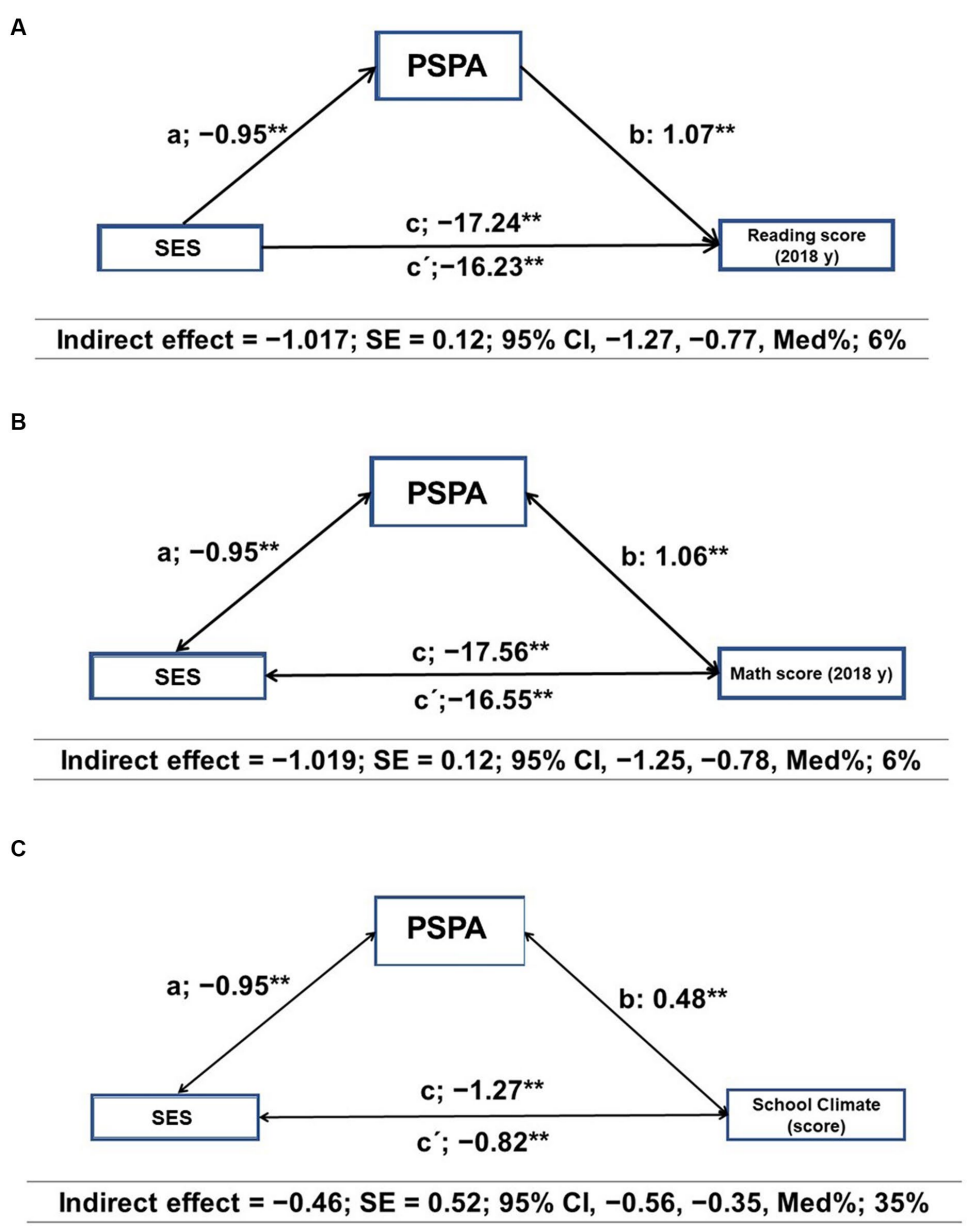
The mediation analysis for the total sample (n=4990 schools). Reference value High-SES. Promoting School Physical Activity (PSA).

The mediation analysis results are shown in Figure 3 for the total sample (*n* = 4,990 schools). PSPA emerged as a mediating variable in the relationship between SES and academic achievement and school climate. In the first regression step (a), SES was inversely related to PSPA (*p* < 0.001). In the second step (c), the regression coefficient of SES in academic achievement was also significant (*p* < 0.001). In the third step, the potential mediator PSPA was positively related to the dependent variable (b) (*p* < 0.001), but when both SES and PSPA were included in the model (c’), the regression coefficient remained statistically significant (*p* < 0.001). Finally, the indirect effect confirmed that PSPA was a partial mediator of academic achievement in reading (IE = 1.017; SE = 0.12; 95%CI, −1.27, −0.77, Med%: 6%) (Figure 3A) and maths (IE = –1.019; SE = 0.12; 95%CI, −1.25, −0.78, Med%: 6%) (Figure 3B). With regard to school climate in the first regression step (a), SES was inversely related to PSPA (*p* < 0.001). In the second step (c), the regression coefficient of SES in school climate was also significant (*p* < 0.0001). In the third step, the potential mediator PSPA was positively related to the dependent variable school climate (b) (*p* < 0.001). Finally, the indirect effect confirmed that PSPA was a partial mediator of school climate (IE = –0.46; SE = 0.52; 95%CI, −0.56, −0.35, Med%: 35%) (Figure 3C).

## 4 Discussion

The study examined two main objectives: (1) determining the association between PSPA and academic achievement and school climate according to SES of schools and (2) exploring the potential mediating role of PSPA in the relationship between SES of schools and academic achievement and school climate. The main results were as follows: (i) the relationship between SES and academic achievement was mediated by PSPA; (ii) there was a positive association between academic changes and PSPA mainly in the low-SES schools; and (iii) there was a relationship between SES and school climate and it was positively mediated by PSPA.

PSPA mediated the association between SES and academic and non-academic achievement, suggesting that promoting PSPA among children could help close the gaps in this unfortunate relationship. Previous studies (42–44) have indicated a relationship between physical activity and academic achievement and have highlighted SES as one of the most relevant mediating variables. Indeed, SES should be viewed as an early risk factor for access to learning, especially because it is often students with inferior economic resources who cannot access regulated physical activity practices and foster those movement skills that have been shown to be directly related to cognitive, emotional, and social development.

One cross-sectional study reported a weak link between PA and academic achievement, necessitating further investigation (45). A second study reported that physical fitness mediated the relationship between SES and academic achievement (46). Another study conducted on Chilean schoolchildren indicated that better physical fitness could be deemed a protective social factor related to bridging the cognitive gap associated with school vulnerability (47). Moreover, a longitudinal analysis found that PA during adolescence predicted higher educational levels and SES in adulthood (48). In a complementary way, PSPA through PA intervention may positively affect academic achievement: for example, 36 developed a 3-year cluster-randomized controlled trial of 24 elementary schools to compare changes in academic achievement and fitness in schools that received a PA intervention of 90 min/week of moderate to vigorous physically active academic lessons and reported positive changes in academic scores in maths and reading in elementary schools in northeast Kansas. Similarly, a study of a 20-week PA intervention among socioeconomically deprived schoolchildren (aged 8–13 years) in South Africa reported a positive effect on academic achievement and physical activity; furthermore, fit children tended to exhibit better concentration than their less fit peers (49). A longitudinal study evaluating the relationship between objectively measured (moderate to vigorous) PA and academic achievement among adolescents showed a long-term positive impact of the former on the latter (50).

In the present study, we found a positive association between academic delta changes and PSPA in reading and maths; in addition, we observed superior academic results in low-SES schools that especially promoted PA. Previous research has shown that PA with increasing aerobic capacity and motor skills could have the potential to improve academic achievement (51). For instance, one systematic review found PA to be associated with cognition (52). Another study noted that PA was decisive in affecting academic achievement, implying that lifestyle behavior is a key factor to consider for improving academic achievement (53). Moreover, a nationally representative sample of Canadian early adolescents found that active and healthy lifestyle behaviors were positively associated with academic achievement (54). Furthermore, a study of Australian schoolchildren reported that following multiple recommendations from PA movement guidelines (by self-report) was valuable for children’s academic achievement and that adhering to screen time guidelines was particularly important (55); hence, PSPA could be a way to promote equal opportunities among children within school environments and support them in adopting healthy lifestyles, increasing PA and decreasing screen time as a result. PSPA could also contribute to reducing social inequalities in academic achievement (56).

Additionally, school climate was mediated by PSPA in relation to low SES. Indeed, previous research has indicated that an appropriate school climate creates a social learning space that favors academic achievement. Accordingly, improving the school climate may increase adolescent students’ PA (57). Moreover, one study assessed the effect of PA interventions on academic achievement and school climate (classroom behaviors) in childhood and analyzed the characteristics of programs that enhance academic performance, finding that PA improved classroom behaviors and benefitted several aspects of academic achievement, especially maths-related skills, reading, and composite scores (22). School climate can positively influence students’ academic achievement, thus potentially reducing the SES gaps and even overcoming existing social and economic barriers (58). One longitudinal study indicated that school climate—especially learning environment—predicted academic achievement in standardized tests (59). A second study reported that school climate dimensions explained the 14.5% variance of academic achievements in subjects who scored below 15th percentile (60). A third study showed that a structural equation model relating academic achievement to eight dimensions of school climate accurately explained 39.6% of the variability seen in school performance (61). A fourth investigation indicated that an unhealthy school climate, involving such problems as bullying and victimization among adolescents, was associated with fewer days of physical education and lower odds of engaging in PA, thereby adding to concerns about the possible negative health consequences of bullying (62). This underscores the need to develop programs targeted at increasing PSPA.

### 4.1 Limitations

Some of the limitations of this study included an inability to determine differences in gender or ethnicity at each school. In addition, school climate was a self-declared perception. Furthermore, it is necessary to continue improving the construction and validation of the questionnaires used by Chile’s Agency of Education Quality to measure PSPA. Moreover, assessing school climate through student surveys presents certain limitations. By contrast, the great strength of this study was its large sample, rendering it highly representative of the Chilean school population.

## 5 Conclusion

The educational establishments that promoted PA during 2018 reported greater changes in academic achievement than those that did not, especially in those schools with large low-SES student cohorts. In addition, the school climate tended to be superior in the former. The improvements in academic achievement were associated with PSPA. Moreover, PSPA was found to play a potential mediating role in the relationship between schools’ SES and academic achievement. In addition, there was an association between SES and school climate, which was mediated by PSPA.

## 6 Practical implications

PSPA can constitute a way of promoting equal opportunities for children within school environments, thereby helping to reduce social inequalities in academic achievement and close SES gaps related to poverty. Given these potential benefits, public policymakers, schools, teachers, and parents are called on to increase efforts to promote PA of students as a tool that can help diminish social disadvantages and inequalities in Chilean schools. Such findings may also prove useful in the discussion, design, and implementation of public policies and other health and education initiatives. This underscores the need to develop actions that foster the development and permanent evaluation of these indicators of PSPA in the Chilean educational system, in addition to generating a space in public policies that seek to promote these national results. For teachers, theoretically, the increase in PA promotion could increase cognitive and class concentration due to several physiological modifications from the hippocampus. Similarly, from a practical point of view, teachers could improve the school climate in class due to the benefits to schoolchildren when these increase their PA amount per day, for example, reducing anxiety, the positive class behavior among mates, among others. For parents, the increased PA of children could help to improve the general wellbeing of children and their mental health, contributing to their integral development, and for students, improved PA levels have a positive impact on physical, psychological, emotional, and social benefits in the present and later stages of life.

## Data availability statement

The raw data supporting the conclusions of this article will be made available by the authors, without undue reservation.

## Author contributions

PD-F: Data curation, Formal analysis, Project administration, Resources, Validation, Writing – original draft, Writing – review & editing. CC-M: Investigation, Validation, Writing – review & editing. DJ-M: Validation, Writing – review & editing. AR-A: Validation, Writing – review & editing. IG-G: Formal analysis, Investigation, Supervision, Writing – review & editing. CA: Formal analysis, Investigation, Validation, Writing – original draft. MG-L: Validation, Writing – review & editing. BC-T: Validation, Writing – review & editing. FC-N: Supervision, Validation, Writing – original draft.
